# Effects of COVID-19 lockdowns on shorebird assemblages in an urban South African sandy beach ecosystem

**DOI:** 10.1038/s41598-022-09099-8

**Published:** 2022-03-24

**Authors:** Jemma Lewis, Jayden Collison, Deena Pillay

**Affiliations:** Department of Biological Sciences, Marine Research Institute, University of Cape Town, Cape Town, 7701 South Africa

**Keywords:** Ecology, Urban ecology

## Abstract

Human pressures are pervasive in coastal ecosystems, but their effect magnitudes are masked by methodological limitations. Government lockdowns associated with the global COVID-19 pandemic can address this gap since lockdowns are effectively manipulations of human presence in ecosystems at scales unachievable otherwise. We illustrate this using a study on shorebirds in an urban South African sandy beach ecosystem. Data collected prior to (2019) and during the COVID-19 (2020) pandemic indicated an inverse relationship between shorebird and human numbers, but this was stronger in 2020. In 2020, human exclusion resulted in a six-fold increase in shorebird abundance relative to 2019. Following easing of lockdowns, shorebird abundance declined by 79.6% with a 34.1% increase in human density. Our findings highlight the sensitivity of shorebirds to recreational disturbance, the potential for current methodological approaches to underestimate repercussions of disturbance and the capacity for COVID-19 lockdowns to refine understanding of human-induced stress in ecosystems.

## Introduction

The establishment of a new geological era by the scientific community—the Anthropocene—reflects the scale and magnitude of human transformation of planet Earth^1,2^. This era is characterised by the twin environmental crises of our time: an unprecedented rate of biodiversity loss and major shifts in the planet’s climate^3–5^. The effects of anthropogenic activities are far-reaching, with at least 70% of the planet’s land surface estimated to have been altered by humans^6,7^ and 66% of oceans being subjected to various human stressors^8^. However, the spectrum of ways in which humans can impact ecosystems, their biotic and abiotic components and multifunctionality (sensu Hector and Bagchi, 2007)^9^ are not fully understood, resulting in inadequate knowledge bases from which to execute management actions in some cases^10^.

Within the marine realm, coastal ecosystems rank amongst the most productive and ecologically significant habitats on Earth—providing key nursery, feeding and refuge functions for a multitude of species, several of which are of commercial and conservation importance^11–14^. From a human perspective, coastal ecosystems also provide access points for marine trade and transport, are rich in subsistence resources and are host to a variety of recreational and cultural activities^15^—features that have long attracted human settlement and facilitated localised population growth^16^. At the same time, densification, urbanisation and resource utilisation are major threats to the ecological integrity of coastal ecosystems^15,16^, with recent assessments indicating some of the most rapid and greatest recent ecosystem declines occurring in the coast^3^.

Of additional concern is that understanding of anthropogenic pressures is widely disparate among coastal ecosystems, with iconic ecosystems receiving more research attention^10^. In sandy beach ecosystems, the problem of research neglect has been raised previously, together with the lack of awareness and appreciation of their ecological relevance on a global scale^10,17^. Management of sandy beaches has been described as having Cinderella status, based on the lower appreciation/awareness of the ecology of these systems in relation to other coastal ecosystems^10^. This is problematic given that sandy beach ecosystems dominate global open-ocean coasts, contributing an estimated 70% to these habitats^17^. Sandy beaches additionally provide socio-economic and ecological goods and services of high value and are key tourist attractions for recreational activity^10,17^. These points therefore suggest that prolonged neglect of sandy beach ecosystems in the context of research on anthropogenic disturbances, is likely to compound their Cinderella status, while risking impairment of ecological multifunctionality in the long-term through ignorance and mismanagement.

A second consequence of the poor research attention afforded to sandy beaches is that consequences of anthropogenic disturbances are not well-understood, which in turn impacts conservation planning and ecosystem management^10,17–19^. Given this, it is possible that human pressure, even when considered to be minor, may have repercussions that are ecologically underestimated or undetected^19^. Such is the case with human recreational activities on sandy beaches, where the existing knowledge base is significant, but knowledge gaps prevail^10,19,20^, with density-dependencies that underlie responses to human activity being an important gap. Specifically, thresholds at which increasing human density alters ecological responses, and the rapidity thereof, are not well understood. This is an issue that needs attention given that (1) sandy beaches are the most frequented of shoreline ecosystems by humans^10^ and (2) knowledge of such thresholds can provide key information on the sensitivity of ecosystem components and processes to stress and management thereof^21^. This assumes great significance given the expected growth of human populations along the coast in future^10^.

Though not exclusive to sandy beach ecosystems, identifying density-dependencies underlying responses to human recreational disturbance is constrained methodologically^22–24^. Specifically, experimental manipulation of human density over meaningful spatial and temporal scales to understand whole-ecosystem responses is unfeasible logistically^23,24^, particularly in urban beaches where prolonged exclusion of humans over large scales can incur significant financial losses, given the dependence of multiple commercial sectors on these ecosystems^10^. Comparisons of beaches with contrasting human density run the risk of ecological responses being confounded by processes unrelated to human presence^23^. The global COVID-19 pandemic however, while being devastating for human lives and livelihoods, has enormous potential to refine understanding of ecological responses in sandy beach ecosystems to human recreational disturbances and associated density-dependencies. This is because government lockdowns instituted to contain viral spread, have created abnormal periods of restricted human mobility, leading to extended and well-regulated time periods in which humans are excluded and permitted in natural ecosystems^25,26^. The pandemic has thus afforded scientists the opportunity to better understand the extent of human pressures on natural ecosystems, including sandy beaches, at a global level and at scales previously unattainable through conventional experimental and comparative approaches^25,26^.

**Figure 1:**
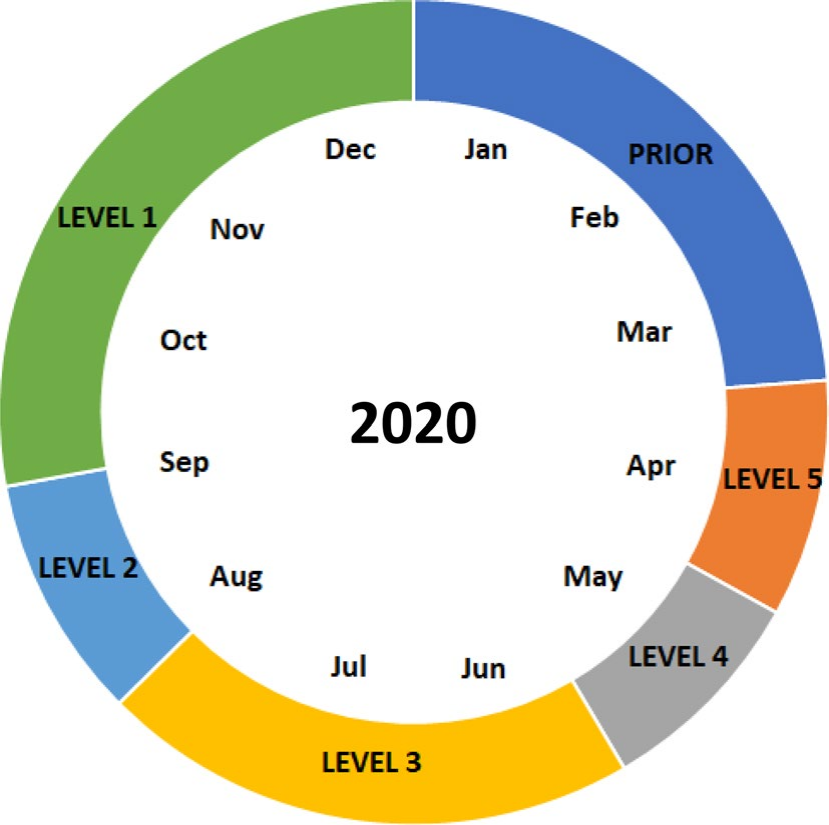
Occurrence and duration of the South African COVID-19 lockdown levels in 2020. January to March: pre-COVID-19 lockdowns (dark blue). Humans were not allowed on beaches in lockdown levels 5 to 3, but access was granted during levels 2 and 1.

**Figure 2:**
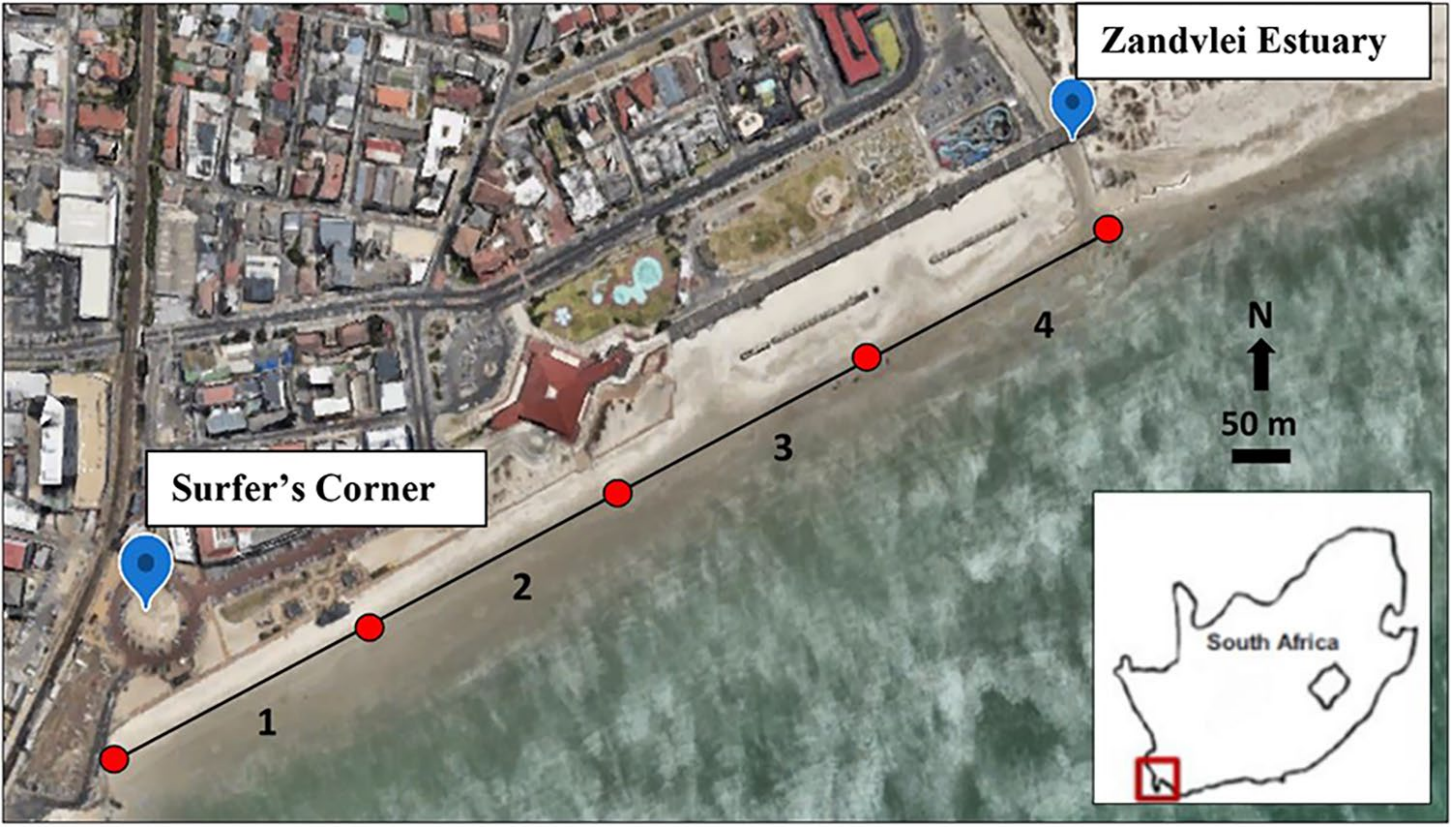
Google Earth image showing a 764 m stretch of Muizenberg beach (black line) from Surfers Corner to the Zandvlei Estuary mouth, including the four spatial zones (191 m each) used for the in situ survey under lockdown levels 3, 2 and 1. Inset: position of Muizenberg Beach within South Africa.

**Figure 3:**
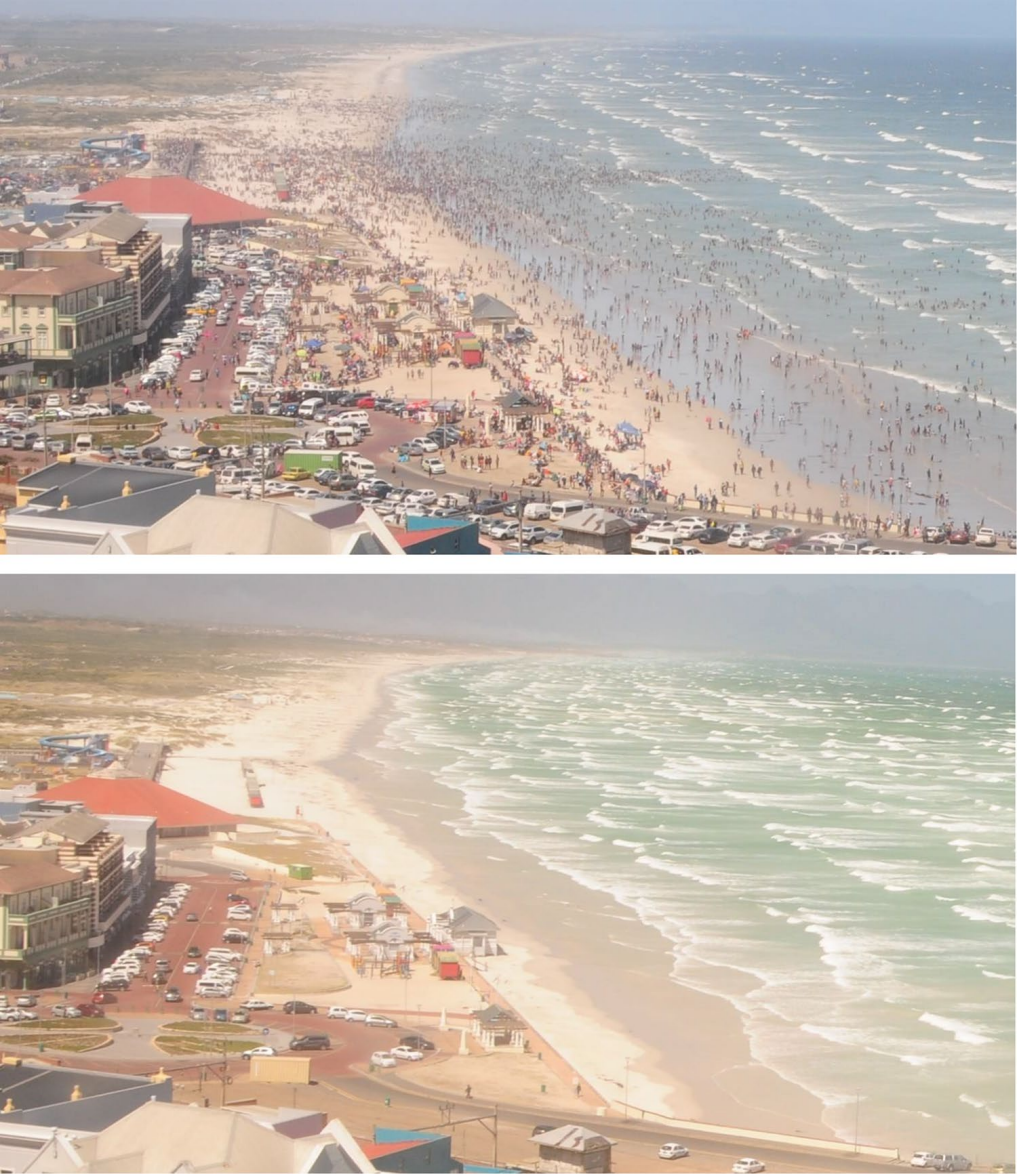
Photographs overlooking Muizenberg Beach on New Year’s Day (1st January;) in (**top**) 2020 (pre-COVID-19) and (**bottom**) 2021 (COVID-19 lockdown. Adjusted lockdown level 3 restrictions imposed to curb viral spread in South Africa’s 2nd wave of infections). Photographs taken at 14H43 courtesy of Associate Professor Coleen Moloney, University of Cape Town.

In South Africa, a national state of disaster was declared on 15th March 2020 in response to the global COVID-19 pandemic^27^. On 27 March, a national lockdown was instituted consisting of five lockdown levels aimed at regulating human movement and curtailing viral spread (Fig. 1), with level 5 imposing the highest restrictions and level 1 the least. Of relevance to this study were the public closures of national beaches during lockdown levels 5, 4, and 3, after which they were reopened (levels 2 and 1)^27–29^. Our study aimed to take advantage of these graded lockdowns and quantify relationships between humans and shorebirds on Muizenberg Beach (Western Cape, South Africa; Fig. 2). Using photographic data, we tested the hypotheses that (1) shorebird and human numbers would be inversely related, but that this relationship would be magnified (steeper slope) in 2020 relative to 2019 (pre-COVID-19 pandemic); (2) shorebird numbers would be greater in 2020 due to extended periods of human absence and (3) greatest bird numbers would occur during lockdown levels 5, 4 and 3, in 2020, when human access to the beach was prohibited. Based on our knowledge of the popularity of Muizenberg Beach and the number of people that visit the beach (see Fig. 3), we expected that increasing human numbers would reduce space availability to birds, thus creating an inverse relationship between bird and human numbers. Noise from people, the presence of dogs and trampling of potential benthic trophic resources were also suspected to contribute to the inverse relationship between bird and human numbers. We expected that the above-mentioned effects would override potential positive effects of increasing human numbers on birds such as food scrap provision. Our hypothesis that the inverse relationship between bird and human numbers would be magnified in 2020 stemmed from the expectation that 2020 would have extended periods of human exclusion (unlike 2019), which would increase space available to birds. From the 2020 data, we aimed to quantify the approximate threshold at which increasing human numbers would rapidly induce a decline in bird numbers, based on the transition from lockdown levels 5, 4 and 3 (humans excluded) to levels 2 and 1 (humans permitted). We additionally conducted in situ surveys of shorebird assemblages during lockdown levels 3, 2 and 1, during the easing of lockdown restrictions. We tested the hypothesis that shorebird assemblage structure would differ between the lockdown levels in response to increasing human numbers from lockdown levels 3 to 1. We hypothesised specifically that these changes at assemblage levels would manifest mainly due to declines in abundance of dominant shorebird species and assemblage metrics (abundance, richness and diversity) from lockdown levels 3 to 1 in response to increasing human numbers.

## Methods

### Study site and sampling design

Muizenberg Beach (34.1087° S, 18.4702° E) is located in Cape Town, on the west coast of South Africa (Figs. 2, 3). The beach is urbanised (Fig. 3), with developments and businesses centred around beach recreation, fishing, surfing and tourism. Our study comprised two components. Firstly, photographic data spanning the period April 2019 through to November 2020 were used to compare bird numbers across five lockdown levels (levels 5 to 1) in 2020 (during the COVID-19 pandemic) with equivalent periods in 2019 (no lockdowns), prior to the pandemic. Secondly, in situ counts of shorebirds on Muizenberg Beach were undertaken in 2020 between lockdown levels 3 and 1 to understand how shorebird assemblage structure changed between the absence (level 3) and presence (levels 2 and 1) of humans. For convenience, we refer to shorebirds in this paper as all birds that were present on the beach at low tide, irrespective of their taxonomic classification.

### Photographic data: 2019 vs 2020

The rapid onset of the nation-wide lockdown in South Africa along with government-imposed restrictions of humans in sandy beaches prevented in situ sampling to test hypotheses pertaining to impacts of COVID-19 lockdowns on shorebirds. We therefore opportunistically made use of photographs taken remotely overlooking Muizenberg Beach (Fig. 3, camera location: − 34.1096937, 18.4660193) that were collected in a separate study focusing on diatom aggregations (Project Leader Associate Professor Coleen Moloney, University of Cape Town). Photographs were taken using standardised methodology prior to the COVID-19 pandemic (2019) and during strict (levels 5, 4 and 3) and eased lockdown (levels 2 and 1) in 2020. These images provided a photographic database of shorebirds frequenting Muizenberg Beach before and during the COVID-19 pandemic, including graded lockdown levels (5 to 1) in the latter period. All photographs used in the study were taken using a Nikon D300 SLR (single lens reflex) camera mounted on a tripod (50 cm) with a Tokina AF 20–35 mm lens and a polarising filter. Photographs were taken during daylight hours every 12 min daily, from a fixed position from the residence of the lead researcher on the diatom aggregation study, overlooking Muizenberg Beach.

Our photographic database comprised photographs taken on three successive days (to account for among-day variability) centred around a spring low tide (± every 2 weeks/month; between 9:00 to 9:30 a.m. per spring low tide). Specifically, we utilised photographs taken before, on and after spring low tide to construct the photographic database. We further constrained the database by using photographs taken only over a four-hour period centred around the low tide (interval ± 30 min) for each day. This generated a total of nine photographs per day per spring low tide. Only shorebird abundance could be accurately determined per photograph—identification of birds to genus or species level was not possible due to low resolution under high magnification. From these photographs, the number of humans was also recorded to relate bird numbers to those of humans. Shorebird and human numbers were quantified only within the first 191 m of the beach within each photograph, starting from the point known as Surfer’s Corner (Fig. 2). Beyond the 191 m mark, identifying birds was difficult due to resolution decline in photographs. Analysis of 2020 and 2019 photographs were temporally randomized (photographs were not analysed chronologically from 2019 to 2020) to prevent unconscious temporal bias when analysing photos.

We undertook two preliminary studies prior to analysis of photographs to refine our sampling design, which involved fixed-mounted web cameras overlooking Muizenberg Beach being monitored at the start of lockdown level 5 (27 March to 23 May 2020). Specifically, photographs from two independent cameras (Cam 1: https://surfemporium.co.za/muizenberg-web-cam/; camera location: − 34.1076647, 18.4709808; Cam2: https://www.wavescape.co.za/tools/webcams/muizenberg-2.html; camera location: − 34.1076035, 18.4705635) were downloaded every hour during daylight hours for 2 weeks. From these photographs, frequency histograms of bird numbers per hour of daylight time were created, which identified peak bird occurrence at spring low tide and few shorebirds at approaching high tide. A second study was initiated involving photographs being downloaded (from the above-mentioned cameras) every 30 min for two hours either side of spring low tide. Results of both preliminary studies indicated that analysis of photographs taken at 30 min intervals over a 4 h period straddling spring low tide over 3 days better captured within- and among-day variability in bird numbers in the preliminary study relative to analysis of photographs at 1 h intervals over 2 h.

### In situ data collection (lockdown levels 3, 2 and 1) in 2020

From June 2020 onwards, level 3 lockdown allowed for in situ counts and identification of shorebirds to be undertaken to supplement photographic data collected between 2019 and 2020. Although lockdown level 3 did not permit human access to beaches, it did allow access to surrounding areas, including public walkways and promenades. We thus made use of the main promenade adjacent to Muizenberg Beach to undertake in situ data collection on three successive days straddling spring low tides from June to November of 2020. Muizenberg Beach was divided into four spatial zones from Surfer’s Corner to the Zandvlei Estuary mouth (Fig. 2), with each zone comprising 191 m of a total 764 m stretch of the beach (Fig. 2). By differentiating the beach into zones, a finer spatial understanding of effects of lockdown levels 3 to 1 on the shorebird assemblage could be gained, through detection of whether changes in shorebird assemblages in response to lockdown levels were zone-specific. This was because each zone was associated with different natural and human features that may have influenced bird responses. For example, there was an intertidal rocky outcrop south-west of Zone 1 and Zone 4 was close to the mouth of the Zandvlei Estuary. Zone 2 was near a public parking lot and Zone 3 was close to a recreational facility. Shorebird identification and counts were conducted on foot at low tide, starting at Surfer’s Corner (zone 1) and ending at the estuary mouth (zone 4). Counts were performed 5 m above the high-water mark, with a clear view of the intertidal zone. The number and species of birds were recorded and counted for each zone with only birds on the substrate being recorded. The shorebird species-pool was limited and species were easily identifiable in the majority of cases. Photographs of unidentifiable species were taken for identification at a later stage using an appropriate field guide^30^.

### Data analysis: photographic data: 2019 vs 2020

Shorebird and human count data for 2019 and 2020 were averaged for each of the 3 days per spring low tide over the 2-year period. A mixed-effects model was used to determine whether bird numbers in Muizenberg Beach were affected by year (2019 vs 2020), human numbers and their interaction. A separate mixed-effects model was run on the 2020 data, to assess effects of lockdown level (5 to 1) on shorebird abundance. The models were constructed in the data analysis platform R^31^ using the ‘‘nlme’ package^32^. Bird abundance was the response variable and human abundance and year were predictor variables for the 2019–2020 dataset. Lockdown level was a predictor variable for the 2020 dataset. ‘Season’ (autumn: March–May, winter: June–August, spring: September–November) and ‘week’ (week of each spring tide) were set as random factors in the models. Residual analyses were used to determine if residuals were normally distributed. Prior to running the models, temporal autocorrelation was evaluated visually using ACF (Autocorrelation Function) plots and quantitatively using Durban-Watson tests. Both tests indicated that the 2019 and 2020 data displayed significant (first order) temporal autocorrelation. To overcome this, the autocorrelative structure of the data were specified in both models (*corAR1*) as part of the tests of main predictor effects^32^.

### In situ data collection (lockdown levels 3, 2 and 1) in 2020

Multivariate analyses were conducted on the in situ abundance data in PRIMER v6 with the PERMANOVA + add-on package^33–35^. Data were 4th-root transformed to downweigh disproportionate contribution of dominant species. The Bray–Curtis similarity index was used to generate similarity matrices, prior to producing non-metric multidimensional scaling (nMDS) plots (999 permutations) to visualise differences in shorebird assemblages among lockdown levels (3 to 1) and zones (1 to 4). A permutational multivariate analysis of variance (PERMANOVA; 2-way crossed) was used to test effects of lockdown level, zone and their interaction on shorebird assemblage structure. Where significant factor effects were identified, a pairwise test was run to identify within-factor differences. A two-way crossed similarity of percentages (SIMPER; factors: lockdown level, zone and lockdown level × zone) analysis was used to identify diagnostic species that contributed most to assemblage differences across factors tested, with a cut-off level of 90%. This procedure was chosen since it provides numerical quantification of species contributions across factors tested rather than qualitative contributions (eg vector overlays in CAP). The DIVERSE routine was used to estimate species richness (S), number of individuals (N), and Shannon–Wiener diversity index (H′) of the shorebird assemblage across lockdown levels and zones.

Analysis of variance (ANOVA) was used to test if lockdown level, zone and their interaction significantly affected diversity measures obtained using the DIVERSE routine (S, N, H’) and abundance of numerically dominant shorebird species as identified by SIMPER to account for 90% of the shorebird assemblage. A residual analysis was performed to determine data normality, followed by log-transformation of the data where deviations from normality were detected. Post-hoc Tukey tests were performed to identify within-treatment differences where relevant. All univariate tests were conducted in R^31^.

### Ethics statement

All data were conducted in accordance with guidelines of the University of Cape Town. Ethics approval for the research was not necessary.

## Results

### Photographic data

**Figure 4:**
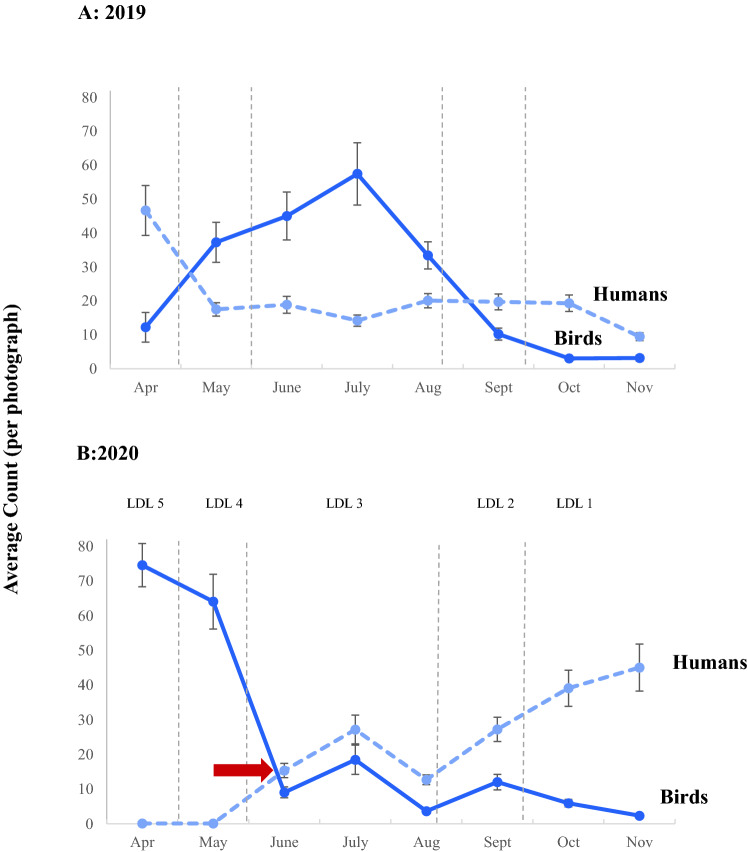
Mean (± 1 SE) shorebird and human abundance at Muizenberg Beach during (**A**) 2019 and (**B**) 2020. Trends are shown for the five lockdown levels (LDL 5 to 1, denoted by horizontal dashed lines) in 2020 and equivalent periods in 2019 (pre COVID-19). The red horizontal arrow in (**B**) shows the confluence of response curves for human and shorebird abundance, from which sensitivity of shorebird to human numbers was estimated during the transition from lockdown levels 5 and 4 to lockdown level 3 in 2020.

Shorebird abundance was negatively related to human abundance and year (2019 and 2020 data) but was positively related to lockdown level (2020 data; *p* < 0.001 for all; Table 1). Shorebird abundance was significantly affected by the interaction between human abundance and year (2019 and 2020 data, *p* = 0.023). During both years, the inverse relationship between shorebird and human abundance was evident but was stronger during 2020 (Fig. 4). Slopes of the regressions of bird abundance against human numbers confirmed this, with the 2020 value (slope = − 0.29) being almost 2 times greater than the 2019 value (slope = − 0.15). During 2020, shorebird abundance was greatest in April (74.56 ± 6.23 per photograph) under lockdown level 5, during which humans were virtually absent from the beach (0.13 ± 0.08 per photograph; Fig. 4B). Although, the highest shorebird abundance over the study was recorded in April 2020, from an inter-annual perspective, abundance was greater in 2019 (Table 1). During 2019, shorebird abundance was greatest over the winter period (May through to August), with shorebird abundance peaking in July (57.46 ± 14.20 per photograph; Fig. 4A). In contrast, the 2019 winter peak in shorebird abundance was not evident in 2020, where, following April and May, decreasing trends were prominent (Fig. 4B). Lowest mean shorebird abundance across both years was recorded in November 2020 during lockdown level 1 (2.36 ± 0.53 per photograph), when human abundance was greatest in 2020 (45.06 ± 6.78 per photograph; Fig. 4B). The 2020 decreasing trend in shorebird abundance over time also explains the significant positive relationship recorded between lockdown level and shorebird abundance (Table 1). Based on the confluence of shorebird and human abundance curves in 2020 (Fig. 4B), we estimated that average shorebird numbers declined by 79.63% (relative to the start of lockdown level 5) with an increase in average human numbers by 34.18% (relative to the end of lockdown level 1) at the transition of lockdown level 4 to 3.

**Table 1:** Mixed effects model outputs testing effects of human abundance, year and lockdown level (LDL) on shorebird abundance over a 2-year period (2019 and 2020) in Muizenberg beach.

Dataset	Predictor	DF	Estimate	SE	t-value	*p*-value
2019–2020	(Intercept)	89	4.02	0.04	22.87	** < 0.0001**
	Humans	89	− 0.07	0.01	− 5.88	** < 0.0001**
	Year (2019 > 2020)	89	− 0.57	0.03	− 3.32	** < 0.001**
	Humans × year (2020 > 2019)	89	− 0.24	0.01	− 2.3	**0.023**
2020	(Intercept)	43	0.42	0.09	2.89	** < 0.001**
	LDL	43	0.69	0.01	11.74	** < 0.0001**

An interaction between year and human abundance is included in the model for the 2019 and 2020 data. LDL results are specific to the 2020 dataset only. Significant *p*-values are shown in bold. ‘Season’ and ‘week’ were included as random factors in both models.

### In situ data collection (lockdown levels 3, 2 and 1) in 2020

A total of seven bird species were identified on Muizenberg Beach during lockdown levels 3, 2 and 1. However, the kelp gull *Larus dominicanus* and Hartlaub’s gull *Chroicocephalus hartlaubii* were most numerically dominant (Supplementary Table S1). PERMANOVA indicated a significant difference in shorebird assemblage structure among lockdown levels (*p* = 0.015) and zones (*p* = 0.001) during the 2020 period spanning lockdown levels 3 to 1 (Table 2). For lockdown level, shorebird assemblages were statistically distinguishable between levels 3 and 2 (*p* = 0.009; Table 2). For zones, zone 1 differed from zones 2, 3 and 4 (*p* < 0.02) and zone 2 was distinct from zone 4 (*p* = 0.01; Table 2). SIMPER indicated that *L. dominicanus* contributed most to assemblage structure within all three lockdown levels (72–93%; Table 3). *C. hartlaubii* contributed 26.17% to the assemblage within level 2 and 12.85% within level 1 (Table 3). As with lockdown level, *L. dominicanus* was again the dominant shorebird across all spatial zones (77–99%) Table 3). *C. hartlaubii* only made contributions to dominant species in zone one (21.67%) (Table 3).

**Table 2:** Results of multivariate PERMANOVA testing effects of lockdown levels (LDL), zones and their interaction on shorebird assemblage structure in Muizenberg beach.

Predictor	DF	SS	MS	Pseudo-F	*p*-value	Pairwise testing
LDL	2	3835	1918	3.114	0.015*	3 ≠ 2
Zone	3	15,274	5091	8.268	0.001***	1 ≠ [2,3,4]. 2 ≠ 4
LDL × zone	6	1393	232	0.377	0.973	

Significant *p*-values are shown in bold (*p* < 0.05*, *p* < 0.001***). Results of pairwise testing are shown in the far right column in cases where significant predictor effects were detected. Data from 2020, lockdown levels 3-1, were used in the analysis.

**Table 3:** Results of SIMPER analysis showing shorebird species contributions (90% cut-off limit) to average dissimilarity among lockdown levels (A) and zones (B) in Muizenberg Beach.

A: Lockdown level						
Taxon	LDL	Abundance	Similarity	Similarity/SD	Contribution (%)	Cumulative (%)
*L. dominicanus*	3	1.23	45.94	1.33	93.08	93.08
*L. dominicanus*	2	1.12	30.21	1.10	72.03	72.03
*C. hartlaubii*	2	0.80	45.94	0.60	26.17	98.20
*L. dominicanus*	1	1.23	45.94	0.95	87.15	87.15
*C. hartlaubii*	1	1.23	45.94	0.38	12.85	100.00

B: Zones						
Taxon	Zone	Abundance	Similarity	Similarity/SD	Contribution (%)	Cumulative (%)
*L. dominicanus*	1	1.69	51.16	3.70	77.15	77.15
*C. hartlaubii*	1	1.02	14.37	0.79	21.67	98.82
*L. dominicanus*	2	0.81	39.16	0.97	99.10	91.10
*L. dominicanus*	3	0.79	25.85	0.74	92.99	92.99
*L. dominicanus*	4	1.29	46.36	1.32	92.97	92.97

4th-root transformed data from 2020, lockdown levels 3-1, were used in the analysis. Abundance = numerical abundance per species; Similarity = intra-group similarity; Similarity/SD = similarity/standard deviation; Contribution (%) = relative contribution (%) of each species to community structure; Cumulative (%) = joint contribution of species to community structure.

**Figure 5:**
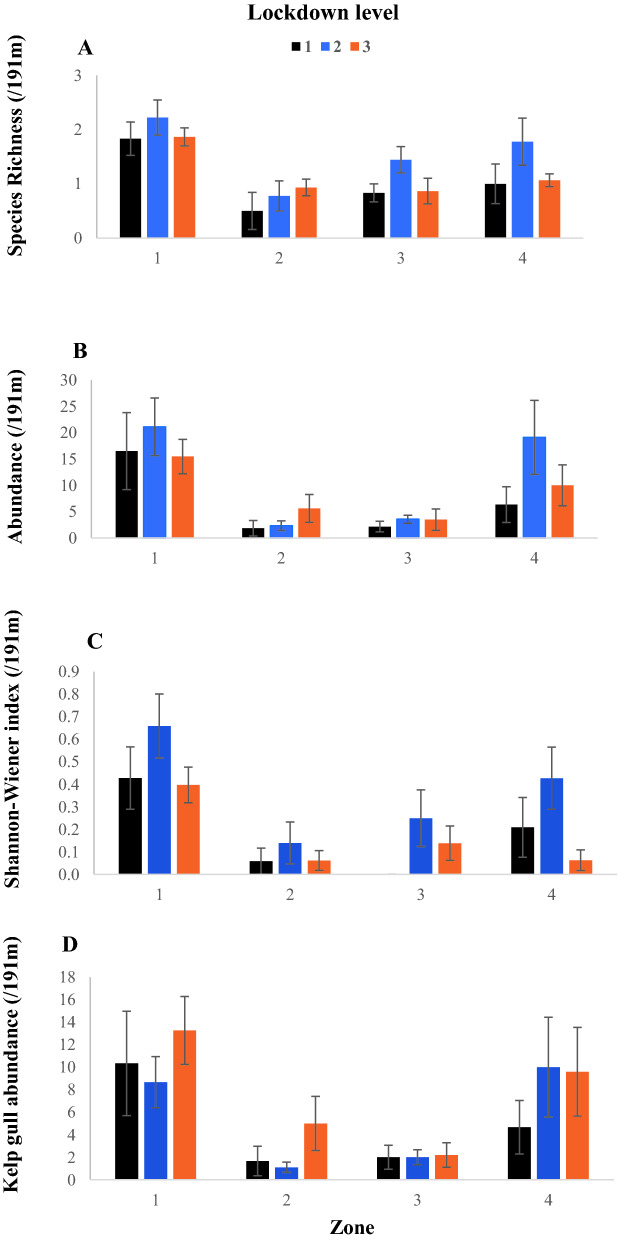
Variation of mean shorebird (± SE) (**A**) species richness, (**B**) abundance, (**C**) Shannon-Weiner diversity and (**D**) mean (± SE) abundance of *L. dominicanus* (± SE) for four zones on Muizenberg Beach during lockdown levels 3 to 1.

ANOVA indicated that the three shorebird assemblage indices differed among zones (*p* < 0.0001; Table 4), with mean values being greater in zone 1 relative to zones 2 and 3 (*p* < 0.001; Table 4, Fig. 5). Additionally, total shorebird richness and Shannon-Weiner diversity were greater in zone 1 than zone 4 (*p* < 0.01; Table 4, Fig. 5). Shannon-Weiner diversity was the only assemblage metric that was significantly affected by lockdown level (*p* = 0.01; Table 4), with levels being greater in level 2 than 3 (*p* < 0.05; Table 4, Fig. 5). The abundance of *L. dominicanus* differed among zones (p < 0.0001), but not lockdown levels, with values generally being greatest in zones 1 and 4 (Fig. 5). Thus, the overwhelming trend recorded for assemblage metrics and dominant species was that variation was more apparent spatially (across zones) than temporally (among lockdown levels).

**Table 4:** Results of ANOVA testing effects of lockdown levels (LDL), zones and their interaction on shorebird assemblage metrics (richness, abundance, Shannon-Weiner diversity) and abundance of Kelp gull (*Larus dominicanus*) in Muizenberg beach.

Predictor	DF	SS	F-value	*p*-value	Tukey post-hoc
**Richness**					
LDL	2	0.74	2.61	0.0780	
Zone	3	4.97	11.70	< 0.0001****	1 ≠ [2,3,4]
LDL × zone	6	0.81	0.96	0.4570	
**Abundance**					
LDL	2	4.18	1.98	0.1430	
Zone	3	48.00	15.17	< 0.0001****	1 ≠ [2,3]. 4 ≠ [2,3]
LDL × zone	6	4.01	0.63	0.7030	
**Shannon-Weiner diversity**					
LDL	2	0.50	5.40	0.0100*	1 ≠ [2,3,4]
Zone	3	1.56	11.23	< 0.0001****	2 ≠ [1,3]
LDL × zone	6	0.21	0.75	0.6080	
***L. dominicanus* abundance**					
LDL	2	1.76	0.95	0.3890	
Zone	3	37.99	13.70	< 0.0001****	2 ≠ 3,4 ≠ [1,2]
LDL × zone	6	0.60	0.60	0.7300	

Significant *p*-values are shown in bold (*p* < 0.05*, *p* < 0.001***). Results of post-hoc Tukey tests are shown in the far right column in cases where significant predictor effects were detected. Data from 2020, lockdown levels 3-1, were used in the analysis.

## Discussion

Graded lockdowns imposed by the South African government to manage the COVID-19 pandemic^27–29^ has afforded us a unique opportunity to quantify shorebird responses to increasing human density in Muizenberg Beach over 8 months in 2020, including a 2-month period of virtual human exclusion. In spite of our study being limited to one beach over 2 years, we were able to take advantage of data collected prior to- (2019) and during the 2020 COVID lockdowns, to better understand a pervasive feature of sandy beach ecosystems (human recreation) that is predicted to intensify in future^10^.

Findings for the 2019–2020 component of our study generally conformed to hypotheses posed. Firstly, shorebird abundance was inversely associated with human abundance and was positively related to lockdown level in 2020. Secondly, shorebird abundance was generally greatest during lockdown levels 5 and 4, when humans were effectively absent from the beach. To contextualise, shorebird abundance was roughly six times greater at the start of lockdown level 5 (2020) than the equivalent period in 2019. Thirdly, lowest shorebird abundance occurred during lockdown level 1 when human abundance was greatest in 2020. Collectively, these findings indicate a strong inverse association between shorebird- and human abundance on Muizenberg Beach and align with results of other studies^36–39^. Cumulatively, our findings, allied with prior research highlight the potential for human recreational activity, particularly at high intensities, to impact shorebird utilisation of sandy beach ecosystems, which may in turn affect ecological functions they provide that contribute to ecosystem multifunctionality.

The inverse relationship that we recorded between human- and shorebird abundance likely manifests through the diverse ways in which recreational activity impacts fundamental processes and ecosystem components, which in turn link ecologically to shorebirds^10,36–40^. Muizenberg Beach is popular for surfing, bait-harvesting and general recreational activities, and it is these activities that likely drive the human-shorebird relationship that we report, particularly in 2020. When carried out under high human densities, such activities can lead to a reduction in space available, rendering the ecosystem less suitable as a substrate for birds^36^. Noise pollution and the presence of dogs may further depress habitat suitability^41^. Repeated trampling of sediment can negatively impact macrofaunal populations, which together with altered sedimentary biogeochemistry (e.g. increased anoxia), can reduce trophic resource availability to shorebirds, with benthic bait-collecting compounding these effects^42,43^. At the start of our data collection in 2020, we were unable to identify shorebird species due to lockdown levels 5 and 4 prohibiting human presence on the beach^27–29^. It is probable though that shorebird assemblages during lockdown levels 5 and 4 were not the same as those we identified between lockdown level 3 to 1 (mainly gulls; Table 3). This is based on research showing that increasing environmental disturbances can induce switches in biotic assemblages to those that can tolerate human activities^44^. Thus, the shorebird assemblages we identified during lockdown levels 3 to 1 is potentially the end-result of the mechanisms highlighted above (space reduction, noise, reduced resource availability) acting on shorebird assemblages in the absence of humans (lockdown levels 5 and 4) following humans being permitted onto the beach.

At an inter-annual level, our data revealed idiosyncratic patterns that raise interesting questions about human-shorebird relationships. In 2019, in the absence of any lockdowns, shorebird abundance rose over the winter period (May–August). Winter peaks in abundance have previously been recorded in the literature^45–47^, including for kelp gulls (*Larus dominicanus*), which were the dominant shorebird in Muizenberg Beach. Specifically, winter abundance peaks for this species have been recorded in sandy beaches in the Eastern Cape, the Swartkops Estuary and Algoa Bay in South Africa (southeast coast)^45–47^. However, the absence of a winter abundance peak in 2020 raises the possibility that the 2019 winter-peak was not seasonal but an opportunistic response to decreased human abundance (see Fig. 4A). In South Africa, coastal ecosystems generally experience greatest human numbers in summer, due to warmer conditions and long end-of-year-vacation periods, based on our observations and experiences.

The second inter-annual trend worth noting in our findings is that shorebird abundance was greater in 2019 than 2020, despite lockdowns being implemented in 2020. This counterintuitive finding is likely due to lockdowns that excluded people from the beach in 2020 (levels 5 to 3) being too short in duration to facilitate increases in bird numbers in 2020 beyond the 2019 level. This is supported by our data showing that humans were excluded from the beach for a total of 2 months (April and May 2020; levels 5-4) out of the 8-month period during which photographs were analysed. It would have been expected at the onset of the study that humans would be excluded from the beach during lockdown level 3^29^, which would have resulted in an additional two and a half months of human exclusion and potentially a higher mean shorebird abundance for 2020. However, it is clear from our data that humans were present on the beach during level 3. On closer inspection, it is evident that human numbers increased even prior to the end of lockdown level 4. In fact, human abundance was greater under lockdown level 3 in 2020 than in the same period in 2019. Such high numbers of humans on the beach despite prohibitions are likely due to a lack of compliance, confusion around regulations and/or ‘covid fatigue’, which describes the propensity of humans to grow tired of COVID-19 regulations^48^. An additional consideration is that human numbers on the beach increased dramatically during lockdown levels 2 and 1, being almost twice the level recorded in 2019 in the same period. The lower 2020 bird count that we recorded is thus likely a product of the short duration of human exclusions in 2020 (lockdown levels 4 and 5) and the magnitude and rate of increase in human numbers thereafter (levels 3-1). Separately, our findings additionally suggest that surrogates (lockdown levels in our case) are unreliable estimators of human presence or abundance and align with findings elsewhere^24^.

The last noteworthy inter-annual trend in our data was the difference in strength of human-shorebird relationships. While the inverse relationship between human and shorebird numbers was evident in both years, it was only during 2020, when humans were excluded from Muizenberg Beach, that the extent of this relationship was revealed. Specifically, in 2020, human exclusion at the start of lockdown level 5 was accompanied by a six-fold increase in shorebird abundance relative to 2019 at the same period. Additional support for the difference in strength of the human-shorebird relationship is the (1) significant interaction recorded between human numbers and year in explaining shorebird abundance and (2) the almost twofold stronger negative relationship (based on regression slopes) between shorebird and human abundance in 2020 vs 2019. These findings suggest that were it not for the COVID lockdowns in 2020, the extent of increasing human numbers on shorebirds may have been masked. However, it must be borne in mind that inter-annual variation may have played some role in the difference in trends recorded for 2019 versus 2020, though we cannot quantify this, given that we only have data for 2 years. Nevertheless, we suggest that when making conservation/management recommendations, decision-makers need to be cognisant of the potential for human effects on sandy beach ecosystems to be underestimated in studies based on variation in human density, in which human exclusion at appropriate spatial and temporal scales is absent^24^. Concerns have been expressed in the past about the failure of studies to consistently detect large-scale changes in sandy beach ecosystems, including those induced by recreational activities^19^. We suggest that such deficiencies may relate in part to the scarcity of true human exclusions in disturbance studies at meaningful scales in space and time.

Findings from the in situ component of our study suggested that shorebird assemblages were negligibly affected by the transition from lockdown level 3 to 1, but that spatial differences among zones were more prominent. The lack of cases in which lockdown levels interacted statistically with zones (Tables 2, 4) further reinforces our conclusion regarding lockdown effects. Shorebird assemblage structure did vary between lockdown levels 3 and 2, due mainly to increasing contributions of *Chroicocephalus hartlaubii* (Hartlaub’s Gull) from level 3 to 2 and the opposite for *L. dominicanus*. Contrary to our hypothesis, differences in assemblage (Shannon–Wiener diversity was the exception) and species metrics were not detected among lockdown levels. This was likely due to the gradient in human abundance being weak among lockdown levels 3 to 1, relative to levels 5 and 4, with there being no virtual exclusion of humans under level 3 lockdown, as would have been expected given government regulations^29^. It is also possible that under lockdown levels 3, 2 and 1, the shorebird assemblage was simplified and comprised species tolerant of human activities^44^. The increase in Shannon–Wiener diversity value from lockdown level 3 to 2 was counter expectation, but likely reflects increased evenness during lockdown level 2, brought on by the declining dominance of *L. dominicanus* and a greater contribution of *C. hartlaubii*.

Taken in its entirety, our findings provide valuable perspectives on human-shorebird interactions in sandy beaches. Based on our 2020 data spanning lockdowns of decreasing severity, our findings suggest that shorebirds are likely to benefit from human-free periods. This benefit is in reality likely to extend across multiple-trophic levels and is unlikely to be shorebird-specific, based on prior research reporting positive organism metrics at lower trophic levels in low human and/or human-free conditions in beach ecosystems^20^. Broadly, our findings attest to the value of using current and future lockdowns associated with managing the global COVID-19 pandemic to provide data on responses of birds and other organism groups to human-free spaces and times^25,26,49^. These human-free conditions can additionally provide invaluable data on sensitivities of ecosystem components and processes to increasing human density^25,26,49^. Data collected during lockdowns can provide better approximations of baseline conditions in sandy beach ecosystems, thereby providing a more meaningful basis for (1) evaluating future ecosystem change in response to human and global change stressors and (2) developing ecosystem restoration programs. This would be central to preventing long-term ecosystem degradation through the shifting base-line syndrome, where successive generations of decision makers/scientists judge the magnitude of change experienced by ecosystem components against increasingly deteriorating conditions over generational time-scales^50^. We also advocate for data emanating from COVID lockdown studies to be used in public education initiatives, so that beach users are made aware of the ways in which recreational activities can influence beach ecosystems. Such initiatives can improve involvement of public stakeholders in management of sandy beach ecosystems, which has been shown to provide cost-effective and sound decision-making, while increasing support for conservation initiatives^51–53^.

Lastly, our findings have shed light on the sensitivity of shorebirds to increasing human numbers, mainly for recreational purposes. By moving beyond binary contrasts of human presence/absence, our work has also shown the magnitude of increasing human numbers on shorebirds, by virtue of the 34.18% increase in human abundance in our study corresponding with a 79.63% decline in bird numbers during the transition from lockdown level 4 to 3 in 2020. This finding is highly relevant considering that our work was based on an urban ecosystem—such systems are thought to have avian communities that are more disturbance tolerant relative to rural or suburban ecosystems^54^. Broadly, our work emphasises the need for environmental managers and city planners to be cognisant of the sensitivity of shorebirds to human recreational activities, even in urban settings, and to develop appropriate management plans in conjunction with scientists and stakeholders^51–53^. It should be noted that bird responses that we recorded in 2020 are unlikely to be driven solely by changing human numbers in Muizenberg Beach. Processes influencing bird assemblages in beaches surrounding our focal study area, including changes in human numbers and behaviour, may also have been influential determinants of trends recorded. We lack the data to comment meaningfully on this, but is an area worth exploring in future studies.

### Concluding perspectives

The global COVID-19 anthropause has been described as the greatest large-scale experiment in modern history. This period has afforded scientists a unique opportunity to refine understanding of the consequences of human activities on Earth’s natural environments^25,26,49^. This is particularly relevant for human-dominated ecosystems such as sandy beaches, which are arguably the most utilised of Earth’s ecosystems for recreational purposes. In the absence of the COVID-19 anthropause, it is doubtful whether human exclusions could be carried out at scales that would allow meaningful detection of responses to human recreational disturbance. Our findings broadly attest to the points raised thus far, illustrating not only the potential for conventional approaches to underestimate human effects in sandy beaches, but also the sensitivity of shorebirds to human recreation and the magnitude of human influence. We hope that our findings stimulate further research on human recreational effects on sandy beach ecosystems, particularly with a view towards quantifying disturbance sensitivities and response thresholds of fundamental processes that drive multifunctionality in these heavily utilised, yet highly significant coastal ecosystems. We suggest that this is an imperative, given the exponential human population growth expected in the future, particularly along the coast, and the increasing demand predicted on sandy beach ecosystems from recreation, tourism and commercial sectors^10,18^. At its broadest level, our work dovetails with prior calls for scientists to capitalise on current and future COVID lockdowns to refine our understanding of human-nature interactions^25^, so that ecosystems and socio-ecological services provided can be sustainably utilised in the future.

## Supplementary Information

Supplementary Table S1.
