# Supplementary material for: Effects of COVID-19 lockdowns on shorebird assemblages in an urban South African sandy beach ecosystem
Source: Sci Rep. 2022 Mar 24;12:5088. doi: 10.1038/s41598-022-09099-8 (PMC8943502; doi:10.1038/s41598-022-09099-8)
Supplement: Supplementary file 1 — Supplementary Table S1. [file 41598_2022_9099_MOESM1_ESM.docx]

Supplementary Table Captions

Supplementary Table S1: Abundance (mean ± SE) of shorebird species recorded in *in situ* counts during lockdown levels 3 to 1, Muizenberg Beach.

| Common name | Genus and species | Abundance (/764m) |
| --- | --- | --- |
| Kelp gull  Hartlaub’s gull | *Larus dominicanus*  *Chroicocephalus hartlaubii* | 6.88 ± 0.97  2.51 ± 0.66 |
| Common pigeon | *Columba livia* | 0.29 ± 0.09 |
| Common starling | *Sturnus vulgaris* | 0.15 ± 0.12 |
| Cape cormorant | *Phalacrocorax capensis* | 0.03 ± 0.01 |
| Black oystercatcher | *Haematopus bachmani* | 0.03 ± 0.01 |
| White fronted plover | *Charadrius marginatus* | 0.01 ±0.01 |

Supplementary Table S1
